# The Effects of Diet on the Expression of Male Dimorphic Colouration and Weaponry in a Species of Neotropical Katydid

**DOI:** 10.1002/ece3.72630

**Published:** 2025-12-11

**Authors:** Lewis B. Holmes, Carl D. Soulsbury, Fernando Montealegre‐Z

**Affiliations:** ^1^ School of Natural Sciences Lincoln UK

**Keywords:** alternative reproductive tactics, bush cricket, diet, male dimorphism, nutrition

## Abstract

Male alternative reproductive tactics are taxonomically widespread, with many species showing males with distinctly different phenotypic characters such as body size or weaponry. Several mechanisms can drive the expression of these male morphs, including genetic polymorphism or environmental interactions during development. In insects, multiple male morphs are common in several orders, including Coleoptera, Odonata, and Hymenoptera, but are rare in Orthoptera. This study establishes the presence of two male phenotypic morphs in the bush cricket *Satizabalus jorgevargasi*, a species in which the males display mandibular weaponry, and tests the effects of diet on the expression of male dimorphic characters. Male nymphs were raised under standard conditions until adulthood, whereupon morphological measurements were taken. Males raised under standard conditions showed two male phenotypes on the basis of head size and body colouration—a major morph with larger heads and more colouration, and a smaller and duller minor morph. A second group of male nymphs were housed individually and fed either a high‐protein diet or a high‐carbohydrate diet. Body weight and pronotum length were measured on a weekly basis as the nymphs developed, and once the males had matured, morphological and bioacoustic characters were measured. Diet had a significant impact on these male dimorphic characters, with protein‐fed males having significantly larger heads and mandibles. Additionally, males reared on the high‐protein diet had significantly more regions with colour when compared to carbohydrate‐fed males. Our data parallel that seen in other invertebrate groups, where higher levels of protein during maturation are key to the production of larger male morphs.

## Graphical abstract

Male alternative reproductive tactics are widespread, often involving distinct morphs influenced by genetics or the environment. This study confirms two male morphs in the bush cricket *Satizabalus jorgevargasi*—larger, more colourful majors and smaller, duller minors—and tests how diet affects their development. Results show that protein‐rich diets produce males with larger weaponry and more colouration, highlighting the role of nutrition in shaping male dimorphism.

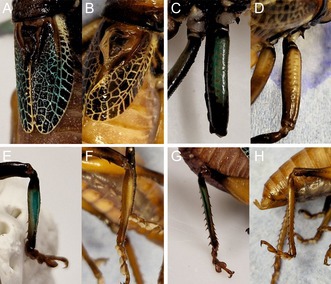

## 1 Introduction

In species with strong sexual selection, males often utilise alternative reproductive tactics (ARTs) to gain reproductive success. ARTs include differences in behaviour, morphology, or both (Cooper 2011; Frayer and Wolpoff 1985; Loyau et al. 2007). These tactics can be either genetically fixed or change throughout an individual's lifetime, and include displays of fitness, sneaking behavior, or physical combat (Kelly 2006; Küpper et al. 2016; Tsubaki 2003). In some taxa, males possess phenotypic variation or multiple morphotypes linked to their tactics, such as ornaments and enlarged weaponry (Emlen 1997a; Loyau et al. 2007; Moczek and Emlen 2000) or female mimicry (Farrell et al. 2013; Griebling et al. 2020). Several ARTs can often coexist within the same population, each maintained through different mechanisms.

Selective processes lead to the evolution of dimorphism within a single sex, either in the form of distinctly different phenotypes and/or behaviours. Male morphs and ARTs can emerge through genetic polymorphisms, interactions between genetics and the environment, and developmental factors. Male ruffs ( *Philomachus pugnax* ) exhibit three genetically determined morphs, with each differing in mating behaviour, plumage, and body size, all controlled via an autosomal polymorphism (Küpper et al. 2016; Lamichhaney et al. 2016; Van Rhijn 1973). By contrast, the mandibular size of the Wellington tree wētā (*Hemideina crassidens*) is determined by the instar at which males mature (8th, 9th, or 10th), representing a plastic response to genetic polymorphisms or environmental factors (Kelly 2004, 2024; Spencer 1995). Environmental conditions, such as diet and climate, can also shape morph expression. Larval nutrition in the damselfly *Mnais costalis* influences whether males develop as colourful fighters or female‐mimicking sneakers (Plaistow and Tsubaki 2000; Tsubaki 2003; Hooper et al. 1999), whereas low temperatures during maturation increase mandible size in broad‐horned flour beetles (*Gnatocerus cornutus*) (Matsumura et al. 2024; Okada et al. 2006).

Diet and nutrition are especially important determinants of ARTs and male dimorphic characters in invertebrates. Many species exhibit condition‐dependent development, where individuals that receive a suitable amount of nutrition develop exaggerated traits, whereas those that are poorly nourished adopt alternate tactics. In dung beetles (*Onthophagus* spp.), males may mature as large horned major morphs that physically compete for mates, or as smaller, hornless minors that employ sneaker tactics (Emlen 1997a; Moczek and Emlen 2000). The controlling factor that determines male morph is the amount of food and parental care the offspring receive (Hunt and Simmons 2000). Offspring that receive more resources emerge as majors and *vice versa* (Hunt and Simmons 2000; Moczek 1999; Moczek and Nijhout 2002). Similar nutrition‐dependent polymorphisms occur in other coleopterans such as *Allomyrina* (Iguchi 1998), *Dorcus* and *Odontolabis* (Iguchi 2013; Matsumoto and Knell 2017). By contrast, fewer studies find such patterns in other insect orders, but include micro‐ and macrocephalic male morphs in the sweat bee, *Lasioglossum hemichalceum* (Hymenoptera) (Kukuk 1996; Wyman and Richards 2003), sneaker and fighter morphs of *M. costalis* (Odonata) (Hooper et al. 1999), and the colour intensity in male *Phymata americana* (Hemiptera) (Punzalan et al. 2008). However, it is important to note that in cases where dimorphism already occurs in the absence of diet manipulation, diet must only be a modulating factor in the likelihood of maturing as a specific morph and is not the sole cause of dimorphism.

Male ARTs are also present within Orthoptera; however, they are typically associated with differences in acoustic signalling rather than weaponry. Many male Gryllidae sing to attract females (Zuk and Simmons 1997), but some males avoid acoustic signalling to reduce parasitism (Walker 1993). They instead adopt purring calls (Tinghitella et al. 2018) or silent satellite tactics (Hissmann 1990; Simmons 1986). In *Gryllus rubens* , wing morphology influences reproductive tactics. Long‐winged males disperse in search of mates, whereas larger, short‐winged males compete aggressively for females. The expression of these phenotypes is controlled by the allocation of resources, which is heritable (Mole and Zera 1993, 1994; Walker and Sivinski 1986). Male dimorphism of weaponry in Orthoptera is rare and has been recorded in only 10 studies (Table 1). Of these 10 studies, a little over half involve tettigoniids, all of which are situated in South America. In these specific tettigoniids, weaponry can occur as enlarged heads and asymmetrical mandibles (*Satizabalus* spp and *Gnathoclita* spp) or as mandibular tusks (*Dicranostomus* spp and *Listrocelis* spp). In these cases, there is variation in the size and shape of the weaponry within males of the same species (Heller and Helb 2021; Holmes et al. 2024; De Souza et al. 2011); however, the cause of this variation is not obvious. The function of this mandibular weaponry may be used in male–male aggression or mate defence (Hugel 2019; Montealegre and Morris 1999), as in *wētās* (Brown and Gwynne 1997; Kelly 2006), and may also serve to convey visual signs of quality. In some genera, such as *Satizabalus*, enlarged heads and mandibles are accompanied by vivid colouration (Holmes et al. 2024), which may vary in intensity or be absent among males. Of these examples, *S. jorgevargasi* is relatively easy and safe to collect and is quick to establish in captivity (Holmes et al. 2024).

**TABLE 1:** List of Orthopteran species where the males display weaponry.

Species	Family	Distribution	Author description	Literature
*Dentoluzara spatulatus*	Phalangopsidae	Colombia	Cadena‐Castañeda and Quintana‐Arias (2024)	Cadena‐Castañeda and Quintana‐Arias (2024)
*Motuweta isolata*	Anostostomatidae	New Zealand	Johns (1997)	Trewick and Morgan‐Richards (2004)
*Motuweta riparia*	Anostostomatidae	New Zealand	Gibbs (2002)	Field and Deans (2001)
*Anisoura nicobarica*	Anostostomatidae	New Zealand	Ander (1932)	Field and Deans (2001)
*Libanasidus vittatus*	Anostostomatidae	South Africa	Kirby (1899)	Field and Deans (2001)
*Libanasa capicola*	Anostostomatidae	South Africa	Péringuey (1916)	Field and Deans (2001)
*Listrocelis angustifrons*	Tettigoniidae	Brazil	Piza (1960)	Fialho et al. (2014)
*Listrocelis atrata*	Tettigoniidae	Brazil	Redtenbacher (1891)	Fialho et al. (2014)
*Listrocelis carinata*	Tettigoniidae	Brazil	Karny (1907)	Fialho et al. (2014)
*Dicranostomus monoceros*	Tettigoniidae	Peru	Dohrn (1888)	Heller and Helb (2021)
*Dicranostomus nitidus*	Tettigoniidae	Peru	Brunner von Wattenwyl (1895)	Heller and Helb (2021)
*Satizabalus jorgevargasi*	Tettigoniidae	Colombia	Holmes et al. (2024)	Holmes et al. (2024)
*Satizabalus sodalis*	Tettigoniidae	Colombia	Holmes et al. (2024)	Holmes et al. (2024)
*Satizabalus huaca*	Tettigoniidae	Colombia	Holmes et al. (2024)	Holmes et al. (2024)
*Gnathoclita vorax*	Tettigoniidae	Brazil	Stoll (1813)	Hugel (2019)
*Gnathoclita izerskyi*	Tettigoniidae	Peru	Gorochov (2018)	Gorochov (2018)
*Gnathoclita laevifrons*	Tettigoniidae	Peru	Beier (1960)	Beier (1960)
*Gnathoclita peruviana*	Tettigoniidae	Peru	Carl (1921)	Carl (1921)

Despite the plethora of studies regarding the impact of nutrition on sexual signalling and nuptial gift quality in Orthopterans (Hall et al. 2008; Hunt et al. 2004; Wedell 1994), there is little research on the effects of nutrition on other male ARTs and dimorphism such as weaponry and colouration, particularly in katydids (Tettigoniidae). Here, we use a species from the Colombian cloud forests, *Satizabalus jorgevargasi* (Orthoptera: Tettigoniidae), where males are macrocephalic with enlarged mandibles and vivid colouration on the tegmina and legs. They will often position themselves at the entrance of a burrow and use their large head to block the entrance. From personal observation, if a rival male attempts to approach the burrow entrance, the defending male will aggressively flare its mandibles and stridulate in an attempt to deter the male. Previous observations hint at the presence of male dimorphism in these traits (Holmes et al. 2024). It is known in a congeneric of this species that mating typically occurs at night with males calling and females actively seeking them out (De Souza et al. 2011; Montealegre and Morris 1999). We first establish the presence of discrete male phenotypes on the basis of mandible size and colouration, then test whether diet modulates existing male phenotypes.

## 2 Materials and Methods

### 2.1 Specimen Housing and Morphological Measurements

A 5th generation colony of *S. jorgevargasi* specimens was housed at the University of Lincoln, UK, in a PHCBI MIR‐154 cooled incubator that cycled around a mean temperature of 16.5°C ± 1.5°C with a 12:12 h light: dark cycle. They were housed in 45cmx45cmx45cm glass Exo Terra vivarium (PT2605; Exo Terra, Rolf C. Hagen Inc., Baie d'Urfé, Quebec) and were routinely misted every 2 days to maintain a humid environment. The substrate consisted of sphagnum moss with access to dark hiding places such as hollowed out tree branches mimicking their natural environment. Specimens were given free access to a standard mixed diet *ad libitum* (Table 2), food was changed out every 2 days.

**TABLE 2:** A nutritional breakdown of the ingredients used to make the three diets. All measurements are in grams (g).

Diet	Composition	Ingredients									
		Oats	Fish flakes	Bird seed	Bee pollen	Calcium powder	Guinea pig pellets	Milk casein	Potato	Total weight	Percentage
Protein	Amount	40	30	20	20	2	120	40	N/A	272	100
	Protein	4.84	10.2	3.2	4.52	0	21	29.2	N/A	72.96	26.82
	Carbohydrate	22.4	0.72	6.8	11.8	0	24.36	4.4	N/A	70.48	25.91
	Fats	3.36	3.48	7.6	0.84	0	5.16	0.72	N/A	21.16	7.78
Carbohydrate	Amount	120	2	15	20	2	40	N/A	100	299	100
	Protein	14.52	0.68	2.4	4.52	0	7	N/A	1.8	30.92	10.34
	Carbohydrate	67.32	0.05	5.1	11.8	0	8.12	N/A	17.5	109.89	36.75
	Fats	10.08	0.23	5.7	0.84	0	1.72	N/A	0.1	18.67	6.24
Standard	Amount	85	5	15	10	2	93	N/A	N/A	210	100
	Protein	10.29	1.7	2.4	2.26	0	16.28	N/A	N/A	32.93	15.68
	Carbohydrate	47.69	0.12	5.1	5.9	0	18.88	N/A	N/A	77.69	36.99
	Fats	7.14	0.58	5.7	0.42	0	3.9	N/A	N/A	17.74	8.45

**FIGURE 1:**
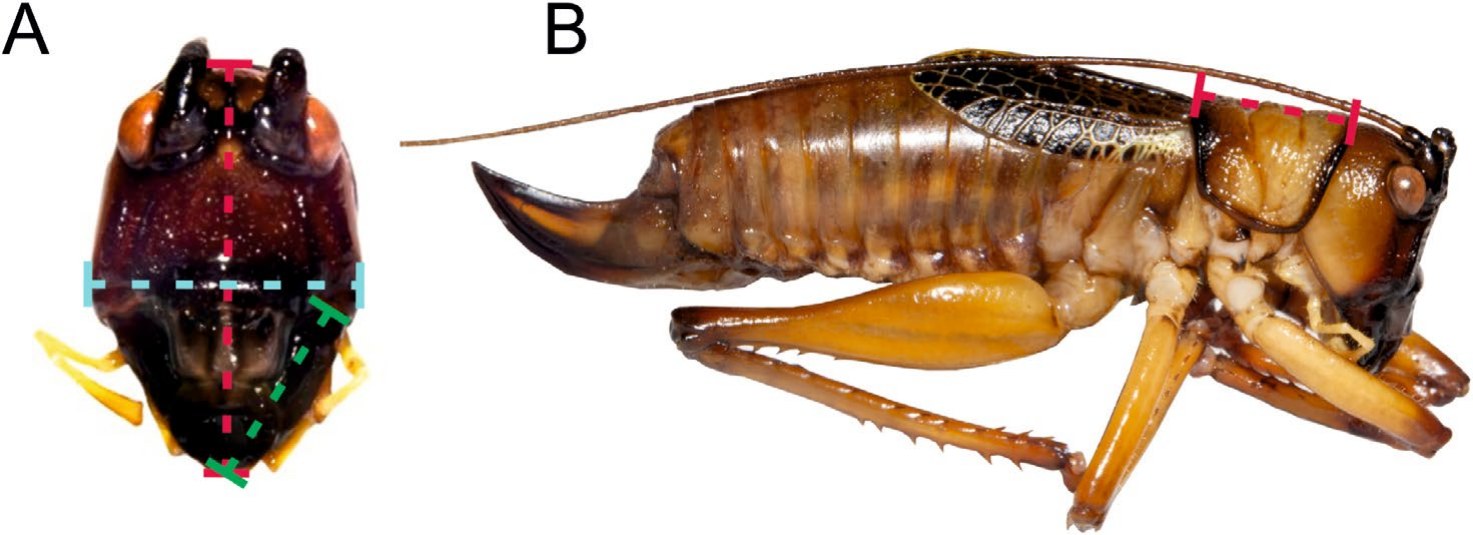
Habitus of *Satizabalus jorgevargasi* showing routes of measurements. (A) Head of *S. jorgevargasi*; Red measurement, head length; Turquoise measurement, head width; Green measurement, mandible length. (B) Side profile of *S. jorgevargasi*; Red measurement, pronotum length.

From this colony, 43 male second instar nymphs were removed and housed in groups of five to maintain the natural effects of population pressure in individual containers within a PHCBI MIR‐154 cooled incubator. However, cage origin was not tracked. It is possible to distinguish males from females at this instar by the presence or absence of the ovipositor under a microscope (4× magnification, Lecia DM750; Leica Microsystems, Wetzlar, Germany). They were misted every day and were provided with their standard mixed diet *ad libitum* (Table 2), with fresh food being added every 2 days. Lighting and temperature were consistent with that of the parent colony. Once they had reached adulthood, morphological measurements were recorded, which included head length (mm), head width (mm), pronotum length (mm), body weight (g), and colouration. Morphological measurements were taken using a pair of digital callipers. Measurements included head length (vertex to labrum), head width (gena to gena), pronotum length, left and right mandible length (Figure 1A,B). Body weight was measured using a Sartorius A 200 S analytical balance scales (Sartorius AG, Göttingen, Germany).

**FIGURE 2:**
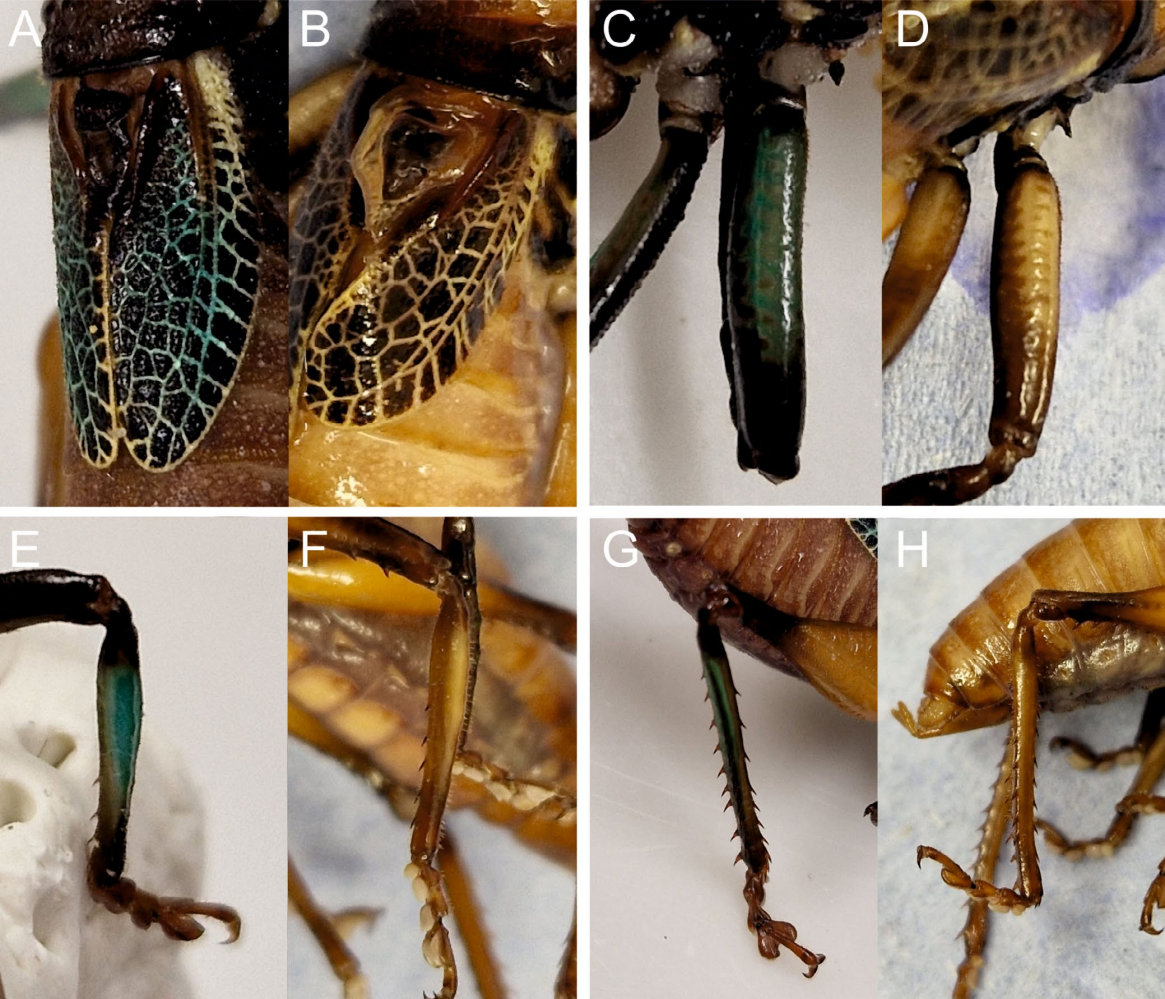
Anatomy of two *Satizabalus jorgevargasi* males exhibiting the presence and absence of colour. (A) Tegmina where verdigris green colouration present. (B) Tegmina where verdigris green colouration is absent. (C) Fore femur with colour present (D) and absent. (E) Mid tibia with colour present (F) and absent. (G) Hind tibia with colour present (H) and absent.

Colouration in the form of a greenish/turquoise presence could be present in 11 possible regions across the animal's body (score 0–11). Those regions were: the left foretibia, forefemur, midtibia, midfemur, hindtibia, the right foretibia, forefemur, midtibia, midfemur, hindtibia, and the tegmen. Colouration was measured on whether the greenish/turquoise hue was present or absent in these areas (e.g., Figure 2). Males that were marked as having 11 for colouration had colour present in every possible region, whereas males that scored 0 were absent of any greenish/turquoise presence.

A further 24 male second instar nymphs were removed from the colony and housed individually, to remove the effects of population pressure, within a PHCBI MIR‐154 cooled incubator. They were then split into two groups of 12; one group was fed only a high protein diet, whilst the other a high carbohydrate diet (Table 2). Each week, the body weight and pronotum length of each individual were measured and recorded. Once the nymphs had reached adulthood, acoustic recordings were taken, and the wing resonance was measured. Following this, several morphological measurements were taken, which included body weight (g), pronotum length (mm), colouration, head length (mm), and head width (mm). Time taken to mature was also recorded. Lastly, the adults were euthanised using a killing jar containing cotton wool soaked with acetone. The mandibles were then dissected out under a microscope (4× magnification, Lecia DM750; Leica Microsystems, Wetzlar, Germany) so that the left and right mandible lengths could be measured (Figure 1A).

### 2.2 Lab Recordings and Wing Resonance

The calls of the *S. jorgevargasi* males that were reared on artificial diets were recorded using an ultrasound sensitive 1/8″ microphone (frequency range 6.5–140,000 Hz, Brüel & Kjær, Nærum, Denmark) coupled with a Brüel & Kjær 2633 preamplifier (Brüel & Kjær, Nærum, Denmark) and connected to a G.R.A.S. 12AA 2‐channel power module (GRAS sound and vibration, Denmark). Amplifier output was connected to PSV acquisition software (Polytec GmbH, Waldbronn, Germany), which used a National Instruments (NI) acquisition board. A high‐pass filter was set at 1 kHz, with a sample frequency of 100 k‐samples/s. Calls were recorded at a temperature of 19°C for a duration of 8 s. Six recordings were taken from each male. Males were placed in a wire mesh cylinder suspended 20 cm away from the recording microphone; they were provided with the correct diet and water for the duration of the recordings. Acoustic analysis of the calls was done using a custom script in MATLAB 2024 (MathsWorks, Natick, USA). Syllable period duration was measured from the start point of one syllable to the start point of the next syllable, in accordance with Baker and Chesmore (2020) using Adobe Audition (Adobe, San Jose, California, United States) (Baker and Chesmore 2020).

Forewing resonance was measured from all 24 *S. jorgevargasi* males using laser Doppler vibrometry (LDV) (PSV‐500; Polytec GmbH, Waldbronn, Germany). The tegmina were held in place using a wax made of 50% beeswax (Fisher Scientific, Loughborough, UK) and 50% colophonium (Sigma‐Aldrich Company Ltd., Dorset, UK). A small amount of the wax was applied to the wing hinge to fix the tegmina in place; the wax was then removed once scanning was complete. Acoustic signals for wing excitation consisted of broadband periodic chirps at 2–60 kHz. These were amplified (Ultrasonic Power Amplifier, Avisoft Bioacoustics, Glienicke, Germany) and transmitted to a loudspeaker (frequency range 1–120 kHz, Ultrasonic Dynamic Speaker Vifa, Avisoft Bioacoustics, Glienicke, Germany) that was positioned 15 cm perpendicular to the tegmen. Speaker output was corrected to be flat in the frequency range (±1.5–2 dB), using a B&K ultrasound sensitive 1/8″ microphone positioned next to the tegmen as a reference signal, and a correction file produced by Python code.

### 2.3 Statistical Analyses

Data analyses were run on RStudio 1.4 using R version 4.4.1 (R Foundation for Statistical Computing, Vienna, Austria). For character data collected from the 43 controlled males, we assessed the modality of each trait distribution using an excess‐mass‐based multimodality test (Ameijeiras‐Alonso et al. 2018, 2021) implemented in the R package *multimode* (version 1.5). We first tested against a unimodal null hypothesis (H_o_: 1 mode) and then against a bimodal null hypothesis (H_o_: 2 modes), using 2000 bootstrap replicates in each case. A significant result for H_o_:1 implies evidence of more than one mode; failure to reject H_o_:2 suggests the distribution is consistent with at most two modes. Excess mass refers to the degree to which the empirical density exceeds a threshold under the null hypothesis of modes. The output was in the form of several kernel density estimate (KDE) plots, which represent a continuous probability density curve that visualises the distribution of the data. A Gaussian mixture model using the package *mclust* (version 6.1.1) was also run for the five traits measured. To test whether traits formed cohesive phenotypes, i.e., how traits covaried, we carried out a principal component analysis (PCA) using the package *FactoMineR*. On the basis of these results (Table S1), a single principal component explained 59% of the variation. We carried out a Gaussian mixture model on this PC1. Finally, since colouration was scored as 0–11 on the presence or absence of turquoise colour across anatomical regions, we ran an additional beta‐binomial model using the package *flexmix* (version 2.3–20) comparing two nested models, with a likelihood ratio test to determine whether a single or two‐component model significantly better explained the data.

For the growth data (body weight and pronotum length) collected from the 24 experimental males, we analysed using linear mixed‐effects models with the package *lmerTest* (version 3.1.3) (Kuznetsova et al. 2017), with male age as a fixed effect. Growth often shows a non‐linear relationship with age, so models with linear, 2nd, and 3rd order polynomials of male age were compared using likelihood ratio tests, with the best model reported in the Results: Section 3. Finally, adult character data (carrier frequency, wing resonance, maturation time, head width and length, mandible width and length, pronotum length, body weight, and colouration) collected from the 24 experimental males were analysed using linear models with diet treatment as the fixed predictor variable, and statistical significance was evaluated using an ANOVA on the fitted models. Figures and plots were generated using the package *ggplot2* (version 3.5.1).

## 3 Results

### 3.1 The Distribution of Character Data

**FIGURE 3:**
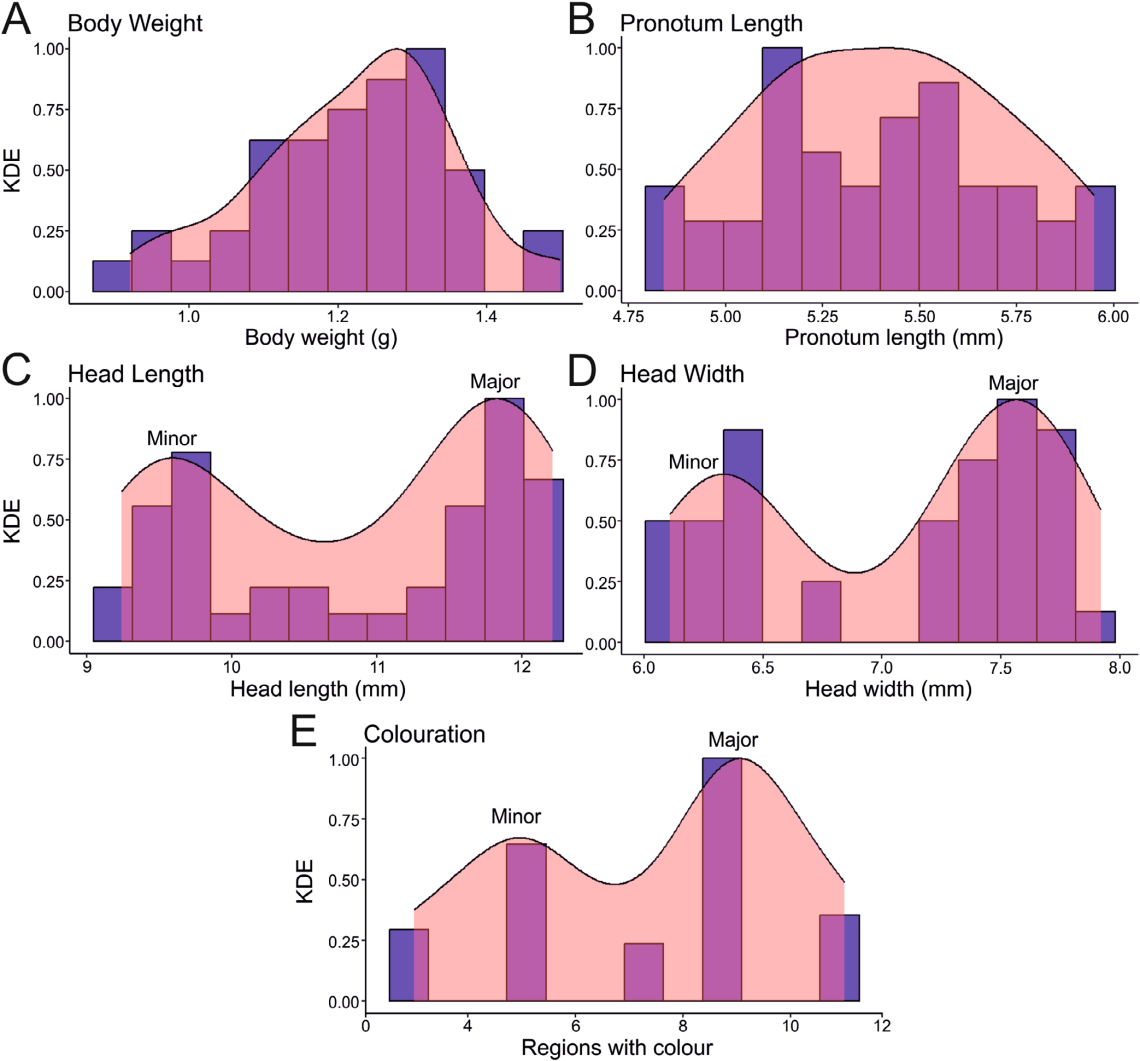
Kernel density estimate plots of character data collected from 43 *Satizabalus jorgevargasi* males. (A) Body weight. (B) Pronotum length. (C) Head length. (D) Head width. (E) Colouration. Panels display a continuous probability density curve that visualises the distribution of the data. KDE; kernel density estimate, Blue bars; frequency of data, Pink area and curves; kernel density estimate.

Body weight (Excess mass = 0.075, *p*‐value = 0.762; Figure 3A) and pronotum length (Excess mass = 0.078, *p* = 0.841; Figure 3B) data did not significantly differ from a unimodal distribution. However, the distribution of head length and head width data differed significantly from a unimodal distribution (Excess mass = 0.201, *p* < 0.001; Excess mass = 0.216, *p* < 0.001), but was not different from a bimodal distribution (Excess mass = 0.087, *p* = 0.093; Excess mass = 0.062, *p* = 0.637; Figure 3C,D). Gaussian mixture modelling of head length revealed two discrete morphs (log‐likelihood = −47.91, BIC = −110.86; *n* = 43), with 19 males classed as minors and 24 as majors. The same was true for head width with 17 males as minors and 26 as majors. For colouration data, the two‐component beta‐binomial model was significantly better than the single component model (LR = 43.04, DF = 2, *p* < 0.001; Figure 3E), indicating the presence of two male colour morphs. Gaussian mixture modelling revealed 16 males were classed as minors and 27 as majors (log‐likelihood = −92.79, BIC = −200.64; *n* = 43). Finally, PCA analysis showed a high correlation between head width, head length, and turquoise colouration, but not with other morphological characters (Table S1), with a single component explaining 59% of the variation (Table S1). Analysis again indicated that males formed two distinct morphs (Figure S1).

### 3.2 The Effect of Diet on Male Growth Rate

**FIGURE 4:**
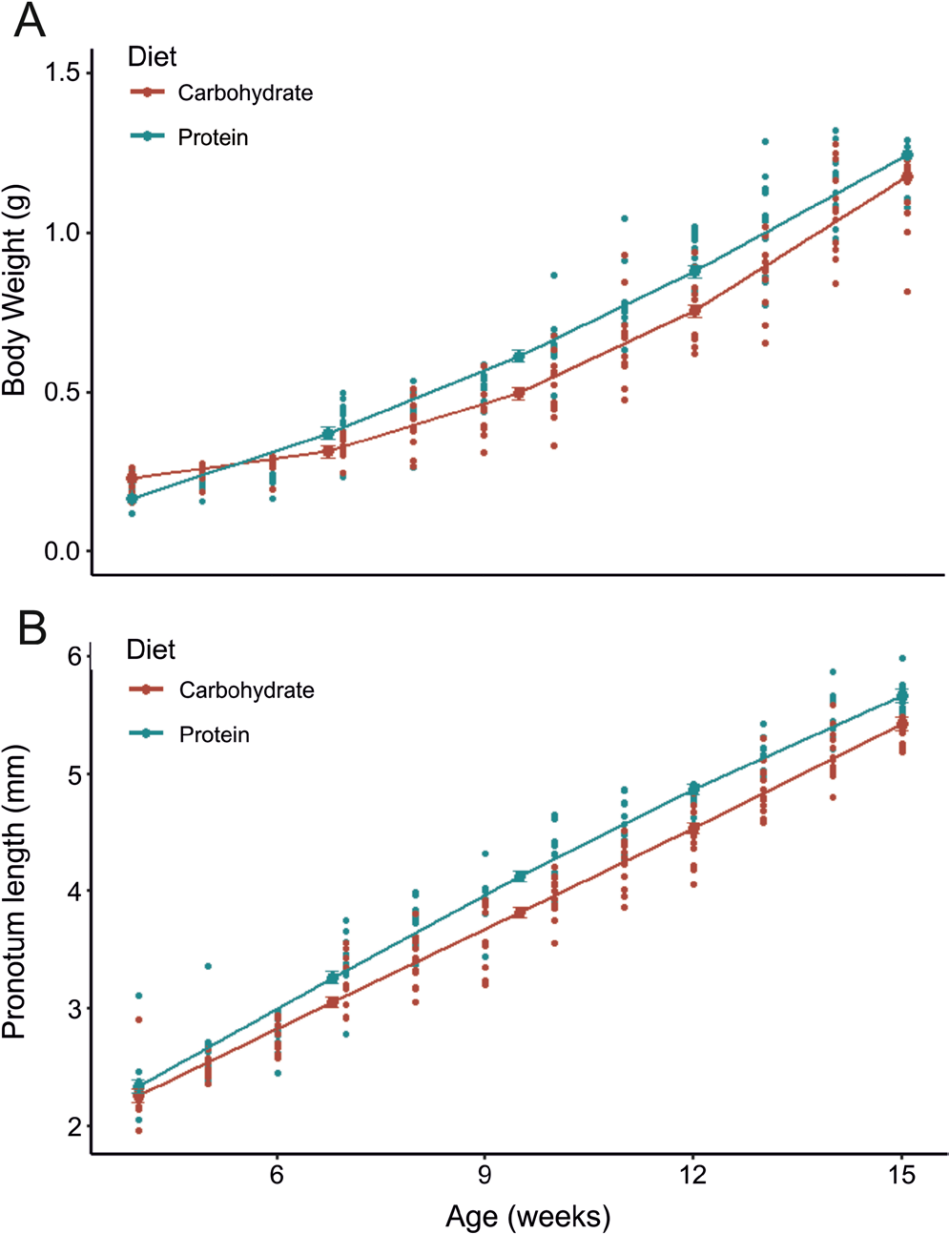
Scatter plots of continuous data collected from 24 *Satizabalus jorgevargasi* males as they mature. (A) Body weight. (B) Pronotum length. Red, High carbohydrate diet. Blue, High protein diet.

Male body weight was significantly affected by the interaction between diet and age^3 (*t* = −3.893, *p* < 0.001) and between diet and age^1 (*t* = 2.807, *p* < 0.001), although no significant interaction was seen between diet and age^2 (*t* = −2.044, *p* = 0.549). In early instars, carbohydrate‐fed males were bigger, but protein‐fed males grew faster (heavier) and to a larger size (Figure 4A). Pronotum length was not significantly affected by diet and age^1 (*t* = 1.904, *p* = 0.058); however, the interaction with pronotum length on diet and age^2 (*t* = −2.462, *p* < 0.01) and diet and age^3 (*t* = −2.536, *p* = 0.011) was significant. Protein‐fed males grew longer pronotums quicker than carbohydrate‐fed males (Figure 4B).

### 3.3 The Effects of Diet on Male Adult Characters

**FIGURE 5:**
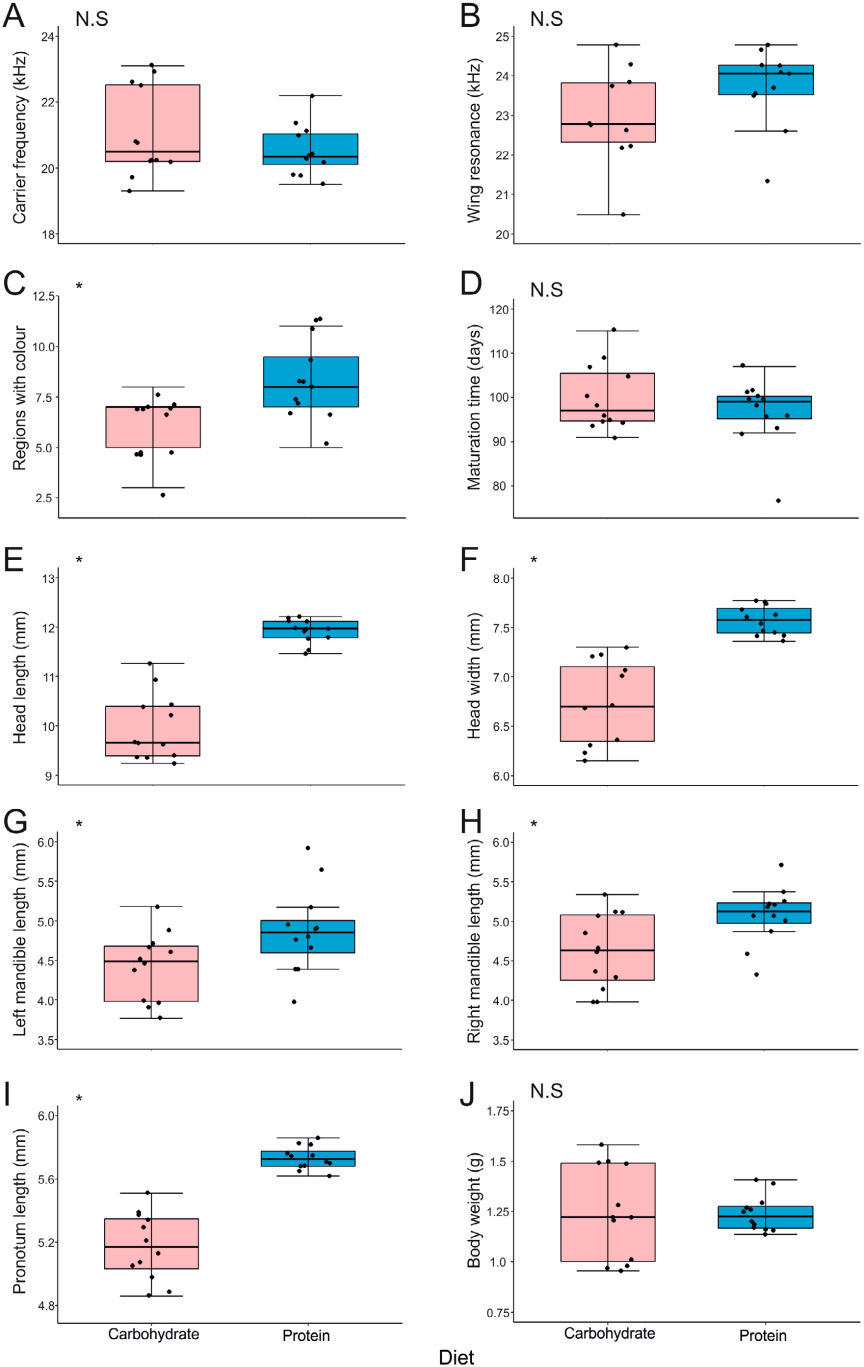
Adult character data collected from 24 *Satizabalus jorgevargasi* males comparing the effects of diet. (A) Carrier frequency (kHz). (B) Wing resonance (kHz). (C) Colouration. (D) Maturation time (days). (E) Head length (mm). (F) Head width (mm). (G) Left mandible length (mm). (H) Right mandible length (mm). (I) Pronotum length (mm). (J) Body Weight (g). Pink boxes; Carbohydrate diet, Cyan boxes; Protein diet, N.S.; no significance, *; statistical significance. Significance was calculated through the use of linear models. For all plots, horizontal lines indicate the median, boxes indicate the interquartile range (IQR), whiskers indicate points within 1.5 IQR, and any data not included in the box and whiskers are shown as outliers (small circles).

There were no significant differences between the two dietary groups with regard to carrier frequency (Estimate = −0.508, SE = 0.448, *t*‐value = −1.135, *p* = 0.269; Figure 5A) and wing resonance (Estimate = 0.499, SE = 0.523, *t* = 0.995, *p* = 0.354; Figure 5B). Additionally, diet had no impact on syllable period duration within the calling song (*x* ^2^(1) = 2.61, *p* = 0.107), but syllable period duration was negatively related to pronotum length (*x* ^2^(1) = 8.84, *p* = 0.02). There was also no significant difference between dietary groups for adult body weight (Estimate = 0.002, SE = 0.072, *t* = −0.034, *p* = 0.973) and maturation time (Estimate = −3.083, SE = 3.033, *t* = −1.016, *p* = 0.327).

However, protein‐fed males had significantly more body regions with greenish/turquoise colour than those fed on the high carbohydrate diet (Estimate = 2.167, SE = 0.692, *t* = 3.132, *p* = 0.005; Figure 5C). Both mandible lengths were significantly affected by diet (Figure 5G,H), the left (Estimate = 0.453, SE = 0.199, *t* = 2.279, *p* = 0.033) and right (Estimate = 0.448, SE = 0.172, *t* = 2.604, *p* = 0.016) mandibles were, on average, longer in protein‐fed males than they were in carbohydrate‐fed males. Similar trends can be seen with head length and width. Protein‐fed males had significantly longer (Estimate = 1.955, SE = 0.205, *t* = 9.519, *p* < 0.001) and wider (Estimate = 0.849, SE = 0.130, *t* = 6.519, *p* < 0.001) heads than those that were fed on the high carbohydrate diet (Figure 5E,F). Lastly, pronotum length was also significantly affected by diet (Estimate = 0.559, SE = 0.064, *t* = 8.691, *p* < 0.001; Figure 5I). Carbohydrate‐fed males had a shorter average pronotum length than protein‐fed males.

## 4 Discussion

### 4.1 An Alternate Male Phenotype

Our data show the presence of two distinct male phenotypes in *Satizabalus jorgevargasi* on the basis of the size of the weaponry and the presence of colour on the legs and tegmina, traits that are likely to influence mating success through their roles in male–male competition. *S. jorgevargasi* is one of the few katydid species in which males employ mate‐guarding (Holmes et al. 2024). This behaviour suggests that male–male competition over burrows and females is likely strong. In this scenario, large mandibles and increased head size may improve performance during aggressive encounters by possibly providing greater bite force or leverage (Goyens et al. 2014; Mills et al. 2016). As the minor males possess smaller heads and less turquoise colouration, they may not be able to displace the major males as easily and would benefit from alternative tactics such as sneaking (Emlen 1997a; Moczek and Emlen 2000). The presence of these two morphs in some traits (head size, mandible length, and turquoise colouration) but not others, particularly acoustic properties, may suggest that although vivid colouration and mandibular size mediate competitive contexts, calling remains a strategy used by both morphs to attract mates.

This parallels scenarios seen throughout a range of taxa where one morph defends a mate and/or territory (usually a major male), whereas the other morph (often a minor) employs sneaker tactics (Emlen 1997b; Moczek and Emlen 2000; Fitzpatrick et al. 2007; Ota et al. 2010; Simmons et al. 1999). In many invertebrate, fish, bird, and other groups, major males are typically larger, more ornamented, and possess weaponry, whilst the smaller minor males sometimes resemble the female in appearance and adopt sneaker behaviours in order to bypass the major males and secure copulations (Emlen 1997a; Fitzpatrick et al. 2007; Hooper et al. 1999; Van Rhijn 1973). As minors are smaller in size, it allows them to invest more heavily in sperm competition and production (Simmons et al. 1999), as found in other taxa with sneaker morphs such as cichlids (Cichlidae) (Fitzpatrick et al. 2007; Ota et al. 2010). Given the guarding behaviour of *S. jorgevargasi* males (Holmes et al. 2024), it is possible that these minor males might be using a similar sneaking behaviour in order to mate and avoid the major males guarding a burrow. The minor males resemble the females with their reduced colouration, although their mandible size remains larger than that of the females. To advance this theory, it would be useful to compare the sperm production of the major and minor *S. jorgevargasi* males, with the expectation being that minor males will have a higher sperm production as they invest less in head size and colouration.

Alternatively, the minor males of *S. jorgevargasi* could adopt a satellite behaviour, which is already documented throughout various Orthopterans (Hissmann 1990; Simmons 1986; Donelson and Van Staaden 2005; Greenfield 1983). This behaviour involves the smaller, non‐singing males positioning themselves near a larger singing male and intercepting females that are attracted by his calling song. In some species, this behaviour is flexible and can be adopted by any male (Hissmann 1990; Simmons 1986). Minor males of *S. jorgevargasi* may adopt this behaviour, allowing them to compete with the larger, major males. Although more research and behavioural observations in the field, such as monitoring a major male's burrow and searching for minor males in the vicinity possibly intercepting females (Bailey and Field 2000; Rowell and Cade 1993), would be beneficial to advance this hypothesis.

### 4.2 Male Growth and Dimorphic Characters

Protein is a key nutrient that influences the growth rates of insects (Punzalan et al. 2008; Lindstedt et al. 2020; Álvarez et al. 2013; Hooper et al. 1999). We found that males fed on the protein‐rich diet had significantly more regions with colour and longer head lengths, widths, and mandible lengths than those reared on the high carbohydrate diet. Given the role of protein in supporting tissue growth (Bowen et al. 1995; Fraser and Rogers 2007), it could be expected that the protein‐fed males would mature more rapidly; however, this was not the case. Growth rates in insects can be influenced by many other environmental and intrinsic factors, including temperature (Hirst et al. 2003), other nutritional dietary components (Cotter et al. 2011; Emlen et al. 2012), and genetics (Nijhout et al. 2014).

Results on the effects of diet on *S. jorgevargasi* males demonstrate that dimorphic traits are strongly condition dependent, relying on protein availability during the nymphal stage to influence weapon size and the presence of colour. These results align with the fact that selected traits are costly to produce and therefore convey fitness and condition (Andersson 1994; Emlen 2008). In many invertebrate taxa, males that receive more nutrition during development typically mature as majors with exaggerated weaponry or ornaments, whereas poorly fed males invest in other tactics (Hunt and Simmons 2000; Kukuk 1996; Moczek 1999; Moczek and Nijhout 2002; Wyman and Richards 2003). Our data are consistent with the literature, with protein‐fed males being more likely to mature as the major phenotype, whilst males reared on a carbohydrate diet were typically smaller minor morphs. However, since bimodality was confirmed in head size and vivid colouration in the 43 males under standard diet condition, diet cannot be the sole modulator of dimorphism; it is instead a factor controlling the likelihood of maturing as a specific morph.

Diet also had a significant impact on the presence of colouration a male expressed, with protein‐fed males expressing more regions with colour than carbohydrate‐fed males. The presence of colour and pigment is costly for an individual to produce and maintain; it should therefore be linked to the individual's fitness and quality, as stated by signalling theory (de I Lanuza et al. 2014; Johnstone 1997; Brenes‐Soto et al. 2017; Van Gossum et al. 2005). The effects of nutrition on male colouration have already been well documented in a range of invertebrate taxa (Álvarez et al. 2013; Punzalan et al. 2008). The correlation between diet and colour presence in *S. jorgevargasi* males suggests that these traits in the legs and tegmina function in mate selection or male–male competition.

Although diet had a significant effect on physical characters such as head size and colouration, it had no impact on the biomechanics of sound production. Both dietary groups produced a similar carrier frequency and had comparable wing resonances. Producing acoustic signals is one of the primary methods of attracting a mate in Orthopterans. It could be expected that nutrition would influence the quality of the calling song produced as this is what males use to convey fitness to and attract a mate (Brown et al. 1996); however, this is not the case. Data collected by Hunt et al. (2004) revealed that in two lab populations of *Teleogryllus commodus*, one reared on high protein and the other on low protein, there was no difference between the carrier frequency of the calling song. Instead, males that were fed the high protein diet spent more time calling for a female and consequently did not live as long as the low protein‐fed males as protein can be used as a metabolic substrate (Hunt et al. 2004; Drake et al. 2012). Additionally, Hall et al. (2008) demonstrated in the Australian ground cricket that the quality of a male's diet had no impact on wing morphology (Hall et al. 2008).

The co‐expression of weaponry and colouration in *S. jorgevargasi* may represent a multimodal signalling strategy in which both traits function together to convey information about male quality. Across taxa, the evolution of coupled signals arises when multiple traits provide complementary information about an individual's condition or fighting ability (Bro‐Jørgensen 2010; Candolin 2003). In *S. jorgevargasi*, the enlarged head and mandibles act as weaponry used during male–male competition, whereas the vivid greenish/turquoise colouration of the legs and tegmina could serve as a visual signal of the male's strength to both rivals and potential mates. The fact that both traits depend on protein intake suggests that they are condition‐dependent indicators of quality, consistent with honest signalling theory (Andersson 1994; Emlen 2008). Therefore, the co‐expression of these traits may enhance the reliability of the signal, with major males displaying both large weaponry and vivid colouration to perhaps deter competitors. Minor males lacking these traits may avoid costly signalling altogether, instead adopting ARTs such as sneaking or satellite behaviour.

## 5 Conclusions

The variation in the rarely seen male dimorphic characters present in *S. jorgevargasi* is great enough for two male phenotypes to be considered on the basis of head length, width, and colouration, likely reflecting alternative reproductive tactics. High protein diets promoted the growth of larger major males, whilst the carbohydrate‐fed males remained smaller and less colourful. However, diet had no effect on maturation time or acoustic characters. Results indicate that both male weaponry and colouration in *S. jorgevargasi* are likely under similar developmental constraints, with diet being one of the key mediators of these traits.

## Author Contributions

**Lewis B. Holmes:** conceptualization (equal), data curation (lead), formal analysis (lead), investigation (lead), methodology (lead), writing – original draft (lead), writing – review and editing (lead). **Carl D. Soulsbury:** conceptualization (supporting), formal analysis (supporting), methodology (supporting), supervision (equal), writing – review and editing (supporting). **Fernando Montealegre‐Z:** conceptualization (supporting), funding acquisition (lead), resources (lead), supervision (equal), writing – review and editing (supporting).

## Funding

This work was supported by the Natural Environment Research Council, NSF DEB‐1937815 – NE/T014806/1.

## Conflicts of Interest

The authors declare no conflicts of interest.

## Supporting information


**Figure S1:** Kernel density estimate plot of each males individual principal component scores. Panel displays a continuous probability density curve that visualises the distribution of the data. KDE, kernel density estimate; Blue bars; frequency of data, Pink area and curves; kernel density estimate.

**Table S1:** Results from PCA analyses showing that head size and colouration covary at the individual level, forming integrated morph phenotypes. Although body weight and pronotum length are unimportant.

## Data Availability

All data and code used in this study has been made available at: Lewis B. Holmes, Carl D. Soulsbury, Fernando Montealegre‐Z (2025). The effects of diet on the expression of male dimorphic colouration and weaponry in a species of neotropical katydid. figshare. Dataset. https://doi.org/10.6084/m9.figshare.30094150.v3.
