# Supplementary figures and images for: The Effects of Diet on the Expression of Male Dimorphic Colouration and Weaponry in a Species of Neotropical Katydid
Source: Ecol Evol. 2025 Dec 11;15(12):e72630. doi: 10.1002/ece3.72630 (PMC12698207; doi:10.1002/ece3.72630)

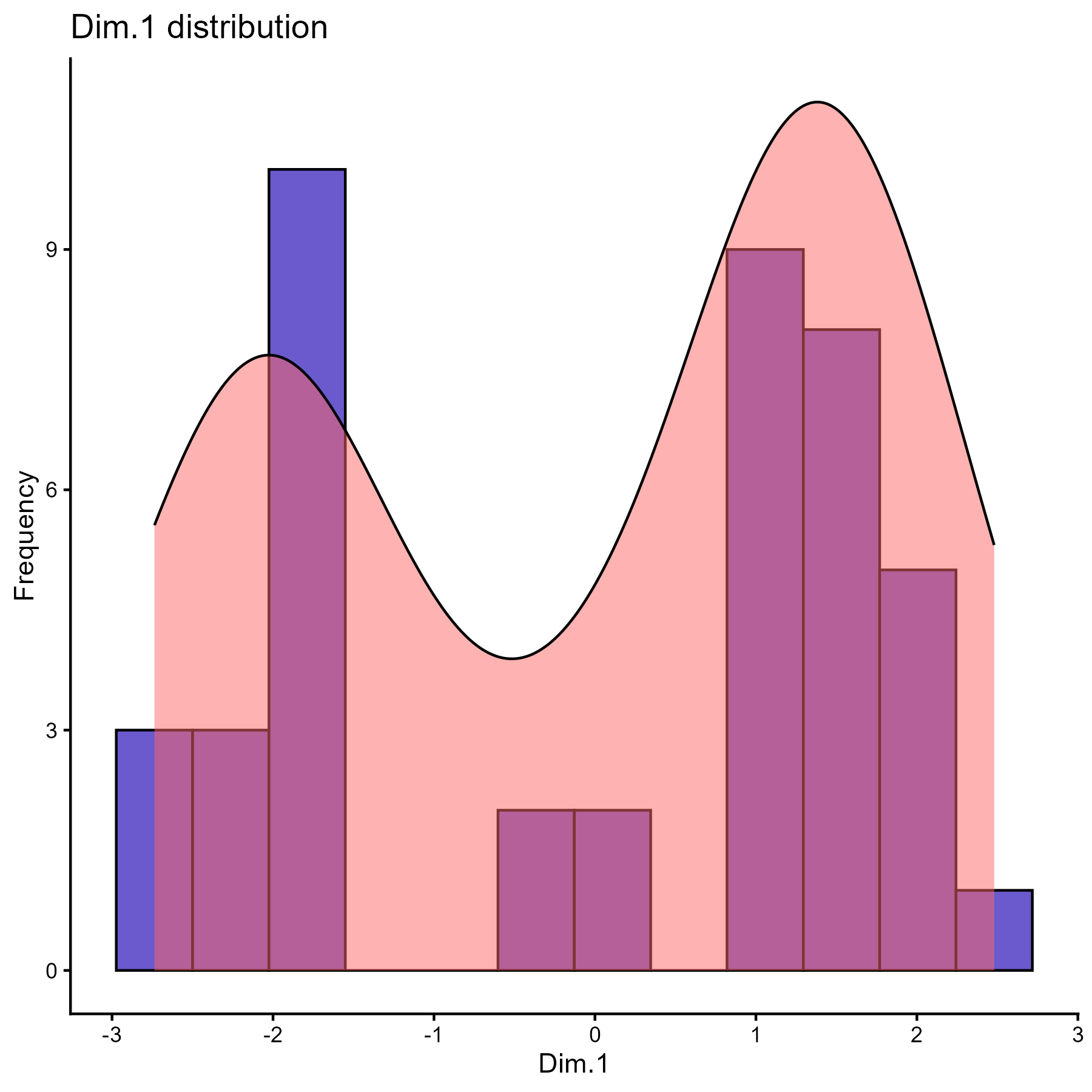

Supplement: Supplementary file 1 — Figure S1: Kernel density estimate plot of each males individual principal component scores. Panel displays a continuous probability density curve that visualises the distribution of the data. KDE, kernel density estimate; Blue bars; frequency of data, Pink area and curves; kernel density estimate. [file ECE3-15-e72630-s002.tiff]
