# Supplementary material for: The Effects of Diet on the Expression of Male Dimorphic Colouration and Weaponry in a Species of Neotropical Katydid
Source: Ecol Evol. 2025 Dec 11;15(12):e72630. doi: 10.1002/ece3.72630 (PMC12698207; doi:10.1002/ece3.72630)
Supplement: Supplementary file 2 — Table S1: Results from PCA analyses showing that head size and colouration covary at the individual level, forming integrated morph phenotypes. Although body weight and pronotum length are unimportant. [file ECE3-15-e72630-s001.docx]

|  | Dim.1 | Dim.2 | Dim.3 | Dim.4 | Dim.5 |
| --- | --- | --- | --- | --- | --- |
| Head length | 0.98 | 0.07 | -0.03 | 0.19 | 0.01 |
| Weight | 0.19 | -0.71 | 0.68 | 0.00 | 0.00 |
| Pronotum | -0.17 | 0.74 | 0.66 | 0.01 | 0.00 |
| Head width | 0.98 | 0.11 | 0.01 | -0.08 | -0.14 |
| Colouration | 0.98 | 0.08 | 0.00 | -0.11 | 0.13 |
| Eigen value | 2.95 | 1.07 | 0.89 | 0.05 | 0.04 |
| Individual proportion of variance | 58.96 | 21.47 | 17.76 | 1.07 | 0.74 |
| Cumulative proportion of variance | 58.96 | 80.43 | 98.19 | 99.26 | 100.00 |

**Supplementary Table S1. Results from PCA analyses** showing that head size and colouration covary at the individual level, forming integrated morph phenotypes. While body weight and pronotum length are unimportant.
